# Association Between Occlusal Wear, Viscoelastic Properties, and Pain in Masticatory Muscles in Individuals With or Without Headache and Neck Pain: A Pilot Cross‐Sectional Study

**DOI:** 10.1155/ijod/7677545

**Published:** 2026-07-30

**Authors:** Divyansh Sinha, Komal Smriti, Prajna P. Nayak, Aditi Chopra, Prabu Raja G., Marwa Khalil, Srikanth Gadicherla

**Affiliations:** ^1^ Department of Periodontology, Manipal College of Dental Sciences, Manipal Academy of Higher Education, Manipal, India, manipal.edu; ^2^ Department of Oral Medicine and Radiology, Manipal College of Dental Sciences, Manipal Academy of Higher Education, Manipal, India, manipal.edu; ^3^ Department of Public Health Dentistry, Manipal College of Dental Sciences, Manipal Academy of Higher Education, Manipal, India, manipal.edu; ^4^ Department of Excercise and Sports Sciences, Manipal School of Health Professions, Manipal Academy of Higher Education, Manipal, India, manipal.edu; ^5^ Department of Oral Medicine, Periodontology, Oral Diagnosis, and Oral Radiology, Faculty of Dentistry, Alexandria University, Champolion St., Azarita, Alexandria 21521, Egypt, alexu.edu.eg; ^6^ Department of Oral and Maxillofacial Surgery, Manipal College of Dental Sciences, Manipal Academy of Higher Education, Manipal, India, manipal.edu

**Keywords:** anxiety, headache, muscle of mastication, occlusal wear, occlusion, orofacial pain, stress

## Abstract

**Objectives:**

Malocclusion and occlusal wear are identified as potential risk factors for altered masticatory muscle function, pain, and temporomandibular joint (TMJ) dysfunction. This study aimed to assess the correlation between occlusal discrepancies, viscoelastic properties, and pain scores in the muscles of mastication—temporalis, masseter, pterygoids, and sternocleidomastoid muscles—in individuals with and without head and neck pain (HNP).

**Methods:**

This pilot exploratory study included 38 participants (10 men and 28 women) divided into 18 patients without HNP (control) and 20 with HNP (case). The following parameters were assessed: severity of tooth wear by the “Individual (personal) Tooth‐Wear Index” by Ekfeldt et al. (1990); viscoelastic properties (tone, stiffness, and elasticity) of masticatory muscles (masseter, temporalis, sternocleidomastoid, lateral pterygoids, and medial pterygoid muscles) using Mytone Pro; and pain scores using the numeric rating scale. Stress and anxiety were assessed using the perceived stress scale (PSS) and the Hamilton anxiety (HAM‐A) scale, respectively. Correlation analyses between occlusal wear and viscoelastic properties of each muscle of mastication were conducted using Pearson’s correlation coefficient. For all analyses, statistical significance was set at *p*  < 0.05.

**Results:**

Participants with HNP exhibited greater mean occlusal wear (8.417 ± 4.54) than those without HNP (6.626 ± 3.72). The frequency and stiffness of the masseter and sternocleidomastoid muscles were significantly greater in individuals with HNP. Pain scores for the muscles of mastication were higher in patients with HNP than in those without HNP. The average pain scores in the muscles of mastication were notably higher in those with HNP (mean = 5.9) than in those without HNP (mean = 3.2). The relaxation time and creep were also reduced in the masticatory muscles of patients with HNP. However, no correlation was observed between masticatory muscle pain and occlusal wear.

**Conclusion:**

Stiffness and relaxation time in the masticatory muscles were greater in individuals with HNP. Occlusal wear affects the viscoelastic properties (tone, relaxation, and stiffness) of the masticatory muscles and is positively correlated with HNP. A multidisciplinary approach involving dentists, neurologists, and physiotherapists is essential for effectively managing patients presenting with headaches, occlusal discrepancies, muscle dysfunction, and craniofacial pain.

## 1. Introduction

**Figure 1:**
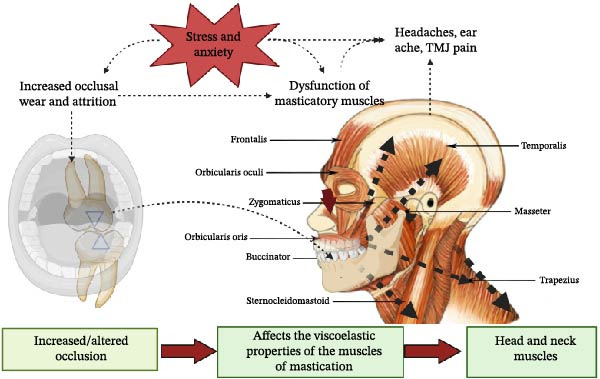
Schematic representation of the link between occlusion, stress/anxiety, headache, and myofascial pain.

The masticatory system is a well‐organized group of craniofacial structures that includes bones, muscles, and neurovascular elements attached to the maxillae, mandible, teeth, temporomandibular joint (TMJ), and cranium [1]. The optimal functioning of the jaw (maxilla and mandible) involves a coordinated movement of all the skeletal and muscular components, including the masticatory muscles, TMJ, teeth, and associated structures of the cranium. The muscles of mastication help move the jaw in different directions and perform a wide range of motions that aid in mastication, speaking, and maintaining the health of the teeth and periodontium [2–4]. The temporalis, masseter, lateral pterygoids, and medial pterygoids are the primary muscles of mastication. The suprahyoid, buccinator, mylohyoid, digastric, omohyoid, infrahyoid, geniohyoid, sternohyoid, thyrohyoid, and sternothyroid are secondary muscles of mastication (Figure 1) [5, 6]. The biomechanical and physiological functions of these masticatory muscles and teeth affect many structures in the head and neck region. The coordinated and synchronous movement of these structures is vital for the effective functioning of the craniofacial apparatus and mastication [7, 8].

Occlusion, defined as the manner in which the teeth of the maxilla and mandible interdigitate, profoundly affects the function of the muscles of mastication [9]. A harmonious contact relationship between the maxillary and mandibular teeth ensures that the forces applied during chewing are distributed evenly to all teeth and even to extraoral structures, such as the masticatory muscles, soft tissue surrounding the teeth (periodontium), and the TMJ. There exits static and dynamic contact relationships between these structures [10]. Maxillary and mandibular teeth modulate the mandibular position and motor control of the masticatory system and are known to affect the muscles of the head, neck, and cervical regions [11]. Occlusal alterations or discrepancies often manifest as attrition, abrasion, alteration of the occlusal table due to cuspal fracture, faulty restoration (over‐contoured or unencountered restorations), premature contacts, significant posterior bite collapse, large centric relation–centric occlusion discrepancies, and unilateral crossbite [12–14]. Hence, any changes in occlusal contact between teeth in terms of abnormal occlusal force, pattern, or manner of occlusion alter the distribution of forces among the teeth, which in turn affects the periodontium, muscles of mastication, and other associated craniofacial structures, such as the TMJ, neck, cervical, and head regions [15–17].

Previous studies have suggested an interrelationship between occlusion discrepancies, dysfunction of the muscles of mastication, TMJ disorders, headaches, neck pain, and craniofacial muscle dystonia [18–22]. TMJ disorder involves the musculoskeletal structures involving the TMJ, muscles of mastication, the cranium, and other craniofacial structures. Masticatory muscle dysfunction, particularly involving the masseter and temporalis muscles, has been strongly associated with headache disorders through the referral of nociceptive inputs and sensitization of trigeminal pathways [23–25]. Previous evidence has found the presence of shared convergent sensory input from the trigeminal nerves entering the muscles of mastication and teeth to the cervical afferents within the brainstem and upper cervical spinal cord [26]. Hence, persistent alterations in occlusion change the functioning of the TMJ, the muscles of mastication, and other craniofacial structures. Alterations in masticatory muscle function and sustained pain from hyperactive or overloaded masticatory muscles can lead to central sensitization and the development or perpetuation of tension‐type headaches and cervicogenic pain [27, 28]. Studies have shown that individuals with TMJ disorders and masticatory muscle dysfunction experience more episodes of primary headache disorders, such as tension headaches and chronic migraines [29, 30]. Patients with TMJ disorders have double the risk of having daily headache patterns, either with or without episodes of migraine [31]. A meta‐analysis found that migraines were more commonly observed in patients with TMJ disorders (40.25%) than tension‐type headaches (18.89%) [25, 32]. A bidirectional association between headaches, TMJ disorders, and masticatory muscle pain, with the link influenced by many environmental, cognitive, emotional, and psychological factors, as well as parafunctional habits such as bruxism, is also explored [33]. Karibe et al. [25] compared myofascial pain symptoms in individuals with and without headaches and found that patients with headaches had higher pain intensity and a higher incidence of sleep difficulties than those without headaches [25]. However, it should be noted that changes in the tone of muscles of mastication are not a direct consequence of headaches; these changes are attributed to the changes in the functioning and physiology of the muscles of mastication modified by other factors, such as occlusal wear, stress, and anxiety [34]. The relationship among stress, anxiety, masticatory muscle, TMJ pain, headaches, and chronic pain disorders affecting the orofacial and cranial regions is complex and interrelated, involving shared psychological and physiological pathways [35–37].

This evidence supports the concept that TMJ dysfunction can influence cervical musculoskeletal function and occlusion, leading to altered head posture, increased cervical muscle activity, and chronic pain in the head and neck [38, 39]. However, most of the evidence is primarily based on the evaluation of electromyographic activity, occlusal force, and palpation‐based muscle alteration and pain assessment, with only limited attempts to measure the changes in the intrinsic viscoelastic properties of masticatory muscles concerning occlusal discrepancies [40–42]. This evidence indicates that most existing studies describe muscle function in terms of activation patterns, tenderness, or thickness rather than intrinsic viscoelastic properties. Previous studies investigating the role of masticatory and cervical muscles in temporomandibular disorders and occlusal pathology have relied mainly on electromyography, bite force analysis, palpation, and morphological assessments, such as assessment of the pain score, muscle thickness, and volume. A study by Della Posta et al. [43] evaluated the viscoelastic properties of the muscles of mastication using Myotome Pro in participants with and without TMJ disorders and found that the viscoelastic properties of the masticatory muscles vary among individuals with TMJ disorders, and this could be used as a tool marker for the early diagnosis of altered TMJ function.

To the best of our knowledge, few studies have simultaneously assessed occlusal wear, masticatory muscle stiffness, and head and neck pain (HNP). Limited evidence exists on the assessment of occlusal changes on the viscoelastic properties (frequency, creep, decrement, relaxation time, and elasticity) of different masticatory muscles. The evaluation of the frequency, creep, relaxation time, stiffness, and elasticity (viscoelastic properties) of the temporalis, masseter, and sternocleidomastoid muscles and their association with occlusal wear severity and psychosocial variables (stress and anxiety) in patients with and without headache or neck pain is also limited. Hence, the present study aims to explore the correlation between the viscoelastic properties (tone, stiffness, and elasticity) and pain scores in masticatory muscles (temporalis, masseter, lateral and medial pterygoids, and sternocleidomastoid muscles) with the severity of occlusal wear in patients with and without HNP. This study also aimed to determine if any correlation exists between the viscoelastic properties of masticatory muscles and occlusal wear and stress and anxiety in participants with HNP. This investigation contributes preliminary evidence regarding the potential association between these factors and may serve as a basis for future longitudinal and mechanistic research studies.

## 2. Methodology

### 2.1. Study Settings

This pilot cross‐sectional exploratory study was conducted at the Manipal College of Dental Sciences, Manipal, in collaboration with Manipal School of Health Professions, Manipal, from August 2019 to February 2020. The study was conducted following the Helsinki Declaration of 1975 (as revised in 2000). The study received ethical clearance from the Kasturba Medical College and Hospital Institutional Ethics Committee with IEC Number: IEC 485 – 2019.

### 2.2. Eligibility Criteria for the Selection of Participants

All participants aged between 20 and 60 years (both males and females) visiting the outpatient Manipal College of Dental Sciences, Manipal, were screened for the presence of HNP based on the following inclusion and exclusion criteria.

#### 2.2.1. Inclusion Criteria

Systematically healthy participants with more than 24 functional teeth in the mouth, except the third molars, were both with and without HNP. The study assessed the self‐reported presence of head and/or neck pain rather than establishing a formal clinical diagnosis.

#### 2.2.2. Exclusion Criteria

• Participants with fewer than 24 functional teeth, except the third molars
• Participant with any systemic disease such as cardiovascular diseases, diabetes mellitus, rheumatoid arthritis, Parkinson’s disease, degenerative or neurological disorders, and any psychiatric disorders (except anxiety and depression)
• Participants taking any medication (analgesics or muscle relaxants)
• Participants who have undergone any dental surgery in the head or neck in the past year
• Participants presenting with any active odontogenic infection, abscess, pain, space infection, or joint disorders
• Participants under any drug/medication affecting their sleep, motor skills, or behavior
• Participants with any trauma, road traffic accidents, or past surgical procedures in the orofacial region.
• Participants with any fracture or ligament injury in the orofacial area
• Participants who were undergoing any treatment, such as physiotherapy or massage, for any pain in the head or neck region
• Mentally and physically challenged individuals

The sample size for the pilot study with 40 participants per group was determined based on an priori power analysis (power = 80%; α = 0.05), using effect size data derived from a prior study by Sierpinska et al. [43] (*p* = 0.009). Participants were recruited through convenience sampling of eligible volunteers who provided written informed consent before undergoing clinical examination and data collection procedures. The consent process included detailed explanations of the study protocols, potential risks and benefits, and voluntary participation rights.

### 2.3. Data Collection

The following demographic data were recorded by two investigators (AC and DS) for all the participants: age (in years), sex (male/female), address, height (in cm), weight (in kg), medical history (presence of any comorbidities), drug history, history of any road traffic accidents/trauma (yes/no), smoking (yes/no), and presence of any oral abusive habits such as the use of areca nut, gutka, paan, supari, and tobacco chewing (yes/no). All participants were asked whether they had experienced any pain in the head and neck region at any time in the past year.

The presence of any pain or discomfort in the muscles of mastication was also assessed by palpating the individual muscle by a specialist (KS) and recorded as dichotomous data (yes/no). The severity of pain in each muscle was recorded via a “numeric rating scale” (score 0–10). The patients were also asked to self‐report and grade the severity of HNP using a numeric rating scale (scores from 0–10) based on pain experienced at any time in the past 5 years from the date of recruitment. A minimum score of 0 was allocated for no pain at all, and 10 was for maximum or intolerable pain. Those participants who reported the presence of HNP were considered cases, and those without HNP at the time were considered the control group. The viscoelastic properties, including muscle tone, stiffness, and elasticity, were determined using Myoton Pro (Myoton AS, Estonia). All measurements were performed at a consistent time of day by the same trained, precalibrated examiner for all participants to avoid intra‐ and interoperator variability.

### 2.4. Assessment of Occlusal Wear

The amount of occlusal wear was assessed using the “Individual (personal) Tooth‐Wear Index” by Ekfeldt et al. [44]. The extent of incisal or occlusal wear for a single tooth was evaluated using the following 4‐point scale: a score of 0 denotes either no occlusal wear or minimal enamel wear; a score of 1 signifies noticeable enamel wear or wear that penetrates the enamel to the dentin at a single location; a score of 2 is assigned when wear reaches the dentin and affects up to one‐third of the crown height; and a score of 3 is applied when dentin wear exceeds one‐third of the crown height, accompanied by significant wear of the tooth restorative material or dental material in the crown and bridgework, affecting more than one‐third of the crown height.

The individual (personal) tooth‐wear index (IA) was calculated from the scores of incisal or occlusal wear for each tooth of that subject based on the following formula: IA = [10 × G1 + 30 × G2 + 100 × G3] / [G0 + G1 + G2 + G3], where G0, G1, G2, and G3 are the number of teeth with scores of 0, 1, 2, and 3, respectively.

### 2.5. Assessment of Myofascial Stiffness

The viscoelastic properties (tone [in Hz], stiffness [in N/m], and elasticity [log decrement]) of the muscles of mastication (masseter, temporalis, and sternocleidomastoid muscles) were determined using a Myoton Pro (Tallinn, Estonia) by a specially trained investigator (PG) who was unaware of the patients’ grouping and severity of occlusal wear, as discussed previously [45]. Myoton Pro was placed vertically at a 90‐degree angle to the external surface of the skin, and the area to be measured was marked. Measurements were recorded twice in a relaxed state, and the average of the two readings was calculated. A tap time of 15 ms and an interval period of 1.5 ms in the multiscan mode were noted. The oscillations induced by MyotonPro on the skin surface were used to determine the viscoelastic properties of specific muscles. For the masseter, the participants were asked to sit upright with their jaw relaxed. The recordings were obtained at the midpoint of the superficial muscle belly, identified during gentle clenching and marked midway between the zygomatic arch and the inferior border of the mandible [46]. For measuring the temporalis muscles, the Mytone was placed on the anterior portion of the muscle belly, superior to the zygomatic arch and ~2–3 cm posterior to the lateral orbital rim [47]. For the sternocleidomastoid muscle, the head of the participants was turned to one side, and measurements were made on the opposite side by placing the myotome at the approximate midpoint between the mastoid process and the sternal/clavicular attachment [48]. The device was connected to the Myoton software, into which the individual participant’s weight, height, nature of dominant hand (right or left), age, and type of muscle were added. The frequency, relaxation, creep, stiffness, and decrement of each muscle (right and left sides) were recorded.

### 2.6. Assessment of Stress and Anxiety

The stress and anxiety levels of each participant were recorded using validated self‐reported questionnaires. The perceived stress scale (PSS)−10 was used to assess stress levels. The PSS‐10 is a self‐reporting tool comprising 10 questions about “how unpredictable, uncontrollable, and overloaded respondents find their lives within the last month” [49]. Each statement on the PSS‐10 ranges from 0 (never) to 4 (very often) on a 5‐point Likert scale. During the assessment, points from 0 to 4 were awarded for each statement, with reversed statements being recalculated, and the scores were determined for each subject. The total score ranged from 0 to 40, with higher scores indicating higher perceived stress levels. The Hamilton anxiety (HAM‐A) scale was used to measure anxiety [50].

### 2.7. Statistical Analysis

The data obtained were compiled in Microsoft Excel (Version 2019, Microsoft, Redmond, Washington, USA) and analyzed using SPSS (Version 26.0, IBM, Chicago, IL, USA). Frequency and percentage distributions were calculated for categorical data, while the mean and standard deviation (SD) were calculated for numerical data. Group comparisons between participants with and without HNP were performed using the chi‐square test for categorical variables and the independent samples *t*‐test for continuous variables (tone [Hz], stiffness [N/m], and elasticity [log decrement] of temporalis, masseter, and sternocleidomastoid muscles). Correlation analyses between occlusal wear and the viscoelastic properties of each muscle of mastication were conducted using Pearson’s correlation coefficient. Multiple linear regressions were performed to predict the severity of occlusal wear from the independent variables. For all tests, statistical significance was set at *p*  < 0.05. Effect sizes (Cohen’s *d* for *t*‐tests, Cramer’s V for chi‐square tests, and standardized β coefficients for regression) with 95% confidence intervals were also calculated.

## 3. Results

A total of 60 participants were screened for the study, of which 40 patients consented to the study and were initially recruited. Of the 40 participants, 38 participants (10 men and 28 women) completed the study. Two participants were excluded as they refused assessment of the masticatory muscles via the myotome. Among all participants, 18 had no HNP (control) and 20 had HNP (case). The mean ages of the participants in the case and control groups were 25.2 and 24.2 years, respectively. The male‐to‐female ratios in the case were 4/14, and in the control group, they were 6/14. The sex and age distributions of the case and control groups are presented in Table 1. The case group showed greater mean occlusal wear (8.417 ± 4.54) than the control group (6.626 ± 3.72). The case group also showed higher individual stress and anxiety scores (21.21 ± 5.64/19.42 ± 5.99) than the control group (15.64 ± 8.99/12.08 ± 6.55). However, these results were not statistically significant (Tables 2 and 3). Table 1 presents the mean occlusal wear, stress, and anxiety scores among participants with and without HNP pain.

**Table 1:** Gender distribution of participants in the case and control groups.

Gender	Data within each gender	Head and neck pain		Total
		Absent	Present	
Male	Count	4	6	10
	% within gender	22.2%	30.0%	26.3%
Female	Count	14	14	28
	% within gender	77.8%	70.0%	73.7%
Total	Count	18	20	38
	% within gender	100.0%	100.0%	100.0%

*Note:* Pearson chi‐square *p*‐value = 0.061 (NS).

**Table 2:** Mean occlusal wear, stress scores, and anxiety scores among participants with and without head and neck pain (HNP).

Outcomes	HNP	Mean	Std. deviation	Std. error mean	Sig. (2‐tailed)
Occlusal wear	Absent	6.626	4.54	1.21	0.195
	Present	8.417	3.72	0.76	
Stress scores	Absent	21.21	5.64	1.50	0.369
	Present	19.42	5.99	1.22	
Anxiety scores	Absent	15.64	8.99	2.40	0.168
	Present	12.08	6.55	1.33	

**Table 3:** Distribution of the individual anxiety and stress levels with the occurrence of head and neck pain (HNP).

Anxiety levels	Category	Without HNP	With HNP	Total
0	Count	7	4	11
	% within anxiety category	38.9%	20.0%	28.9%
1	Count	8	9	17
	% within anxiety category	44.4%	45.0%	44.7%
2	Count	3	4	7
	% within anxiety category	16.7%	20.0%	18.4%
3	Count	0	2	2
	% within anxiety category	0.0%	10.0%	5.3%
4	Count	0	1	1
	% within anxiety category	0.0%	5.0%	2.6%
Total	Count	18	20	38
	% within anxiety category	100.0%	100.0%	100.0%

**Stress levels**	**Category**	**Without HNP**	**With HNP**	**Total**

1	Count	2	4	6
	% within stress category	11.1%	20.0%	15.8%
2	Count	14	14	28
	% within stress category	77.8%	70.0%	73.7%
3	Count	2	2	4
	% within stress category	11.1%	10.0%	10.5%
Total	Count	18	20	38
	% within stress category	100.0%	100.0%	100.0%

*Note:* Pearson chi‐square *p*‐value = 0.69 (NS) for anxiety. Pearson chi‐square *p*‐value = 0.461 (NS) for stress.

Intergroup comparison of the occurrence of HNP with viscoelastic properties (tone, stiffness, and elasticity) of the temporalis, masseter, and sternocleidomastoid muscles showed statistically significant differences between participants with and without HNP. The frequency and stiffness values were significantly higher in the sternocleidomastoid and masseter muscles than in the other muscles in patients with HNP (Table 3). The relaxation and creep mean scores in the sternocleidomastoid and masseter muscles were lower in the HNP group than in those without HNP (Table 4). A comparison of the viscoelastic muscle properties between participants with and without HNP revealed significant differences in the sternocleidomastoid and masseter muscles, with no significant changes noted in the temporalis muscle.

**Table 4:** Intergroup comparison of the occurrence of head and neck pain (HNP) with viscoelastic properties (tone [Hz], stiffness [N/m], and elasticity [log decrement]) of the temporalis, masseter, and sternocleidomastoid muscles.

Viscoelastic property of muscle	Mean		*t*‐Value	Sig (2‐tailed)	Mean difference	Std. error difference	95% Confidence interval of the difference	
	With HNP	Without HNP					Lower	Upper
Temporalis frequency	27.15	26.60	−0.37	0.713	−0.47	1.26	−3.05	2.11
Temporalis stiffness	910.81	883.33	0.18	0.857	12.59	69.33	−128.63	153.80
Temporalis decrement	1.68	1.72	−0.54	0.594	−0.04	0.07	−0.17	0.10
Temporalis relaxation	8.30	8.66	−0.45	0.657	−0.24	0.54	−1.33	0.85
Temporalis creep	0.74	0.77	−0.47	0.642	−0.02	0.04	−0.10	0.06
Masseter frequency	16.86	15.06	3.12	0.004^∗^	2.38	0.76	0.82	3.93
Masseter stiffness	356.94	289.72	2.81	0.008^∗^	95.62	33.99	26.39	164.86
Masseter decrement	1.63	1.55	1.97	0.058	0.18	0.09	−0.01	0.36
Masseter relaxation	17.98	20.93	−2.53	0.017^∗^	−3.39	1.34	−6.13	−0.66
Masseter creep	9.81	11.24	−2.45	0.020^∗^	−1.61	0.66	−2.94	−0.27
Sternocleidomastoid frequency	20.55	18.36	−2.74	0.010^∗^	−0.73	0.97	−2.70	1.23
Sternocleidomastoid stiffness	389.67	327.78	−2.2	0.036^∗^	−22.23	32.92	−89.29	44.83
Sternocleidomastoid decrement	1.24	1.21	0.79	0.435	0.05	0.07	−0.09	0.19
Sternocleidomastoid relaxation	14.52	16.52	2.71	0.011^∗^	0.62	0.89	−1.19	2.44
Sternocleidomastoid creep	1.10	1.28	2.78	0.009^∗^	0.06	0.06	−0.06	0.17

*Note:* Frequency is the rate of muscle contractions, providing insights into muscle function and responsiveness; stiffness indicates the resistance of the muscles to deformation, which can be crucial in understanding muscle tone and health; decrement measures the decline in muscle response over time, which can help assess muscle fatigue and endurance; Relaxation Time assesses how quickly a muscle relaxes after being contracted and reflects on the muscle recovery dynamics; creep indicates the deformation of muscles under sustained load and indicates how muscles adapt to prolonged stress or tension

^∗^Significant *p*‐value < 0.05.

The frequency for masseter muscles was significantly higher in those with HNP than in the control group (mean difference = 2.38, SE = 0.76, 95% CI = 0.82–3.93, *p* = 0.004). Stiffness also increased (mean difference = 95.62, SE = 33.99, 95% CI = 26.39 to 164.86, *p* = 0.008). Conversely, relaxation (mean difference = −3.39, SE = 1.34, 95% CI = −6.13 to 0.66, *p* = 0.017) and creep (mean difference = −1.61, SE = 0.66, 95% CI = −2.94 to‐0.27, *p* = 0.020) were significantly reduced in the HNP group. A borderline effect was noted for the decrement (mean difference = 0.18, SE = 0.09, 95% CI = −0.01 to 0.36, *p* = 0.058). For the sternocleidomastoid muscle, the frequency differed significantly between the groups (mean difference = −0.73, SE = 0.97, 95% CI = −2.70 to 1.23, *p* = 0.010). Stiffness was lower in the HNP group (mean difference = −22.23, SE = 32.92, 95% CI = −89.29 to 44.83, *p* = 0.036). Significant differences were also observed in relaxation (mean difference = 0.62, SE = 0.89, 95% CI = −1.19 to 2.44, *p* = 0.011) and creep (mean difference = 0.06, SE = 0.06, 95% CI = −0.06 to 0.17, *p* = 0.009). In contrast, the temporalis muscle showed no significant differences in frequency, stiffness, decrement, relaxation, or creep (*p*  > 0.05) (Table 4). However, a correlation was not observed between the severity of occlusal wear and the viscoelastic properties of the masticatory muscles. The average pain scores (numeric rating scale) for the muscles of mastication were significantly higher among those with HNP (mean value = 5.9) than among those without HNP (mean value = 3.2) (*p*‐value = 0.0008). No correlation was noted between the pain of the muscles of mastication and occlusal wear (Table S1: Correlation of occlusal wear [score] with severity of muscle of mastication pain [numeric rating scale]). Multiple regression was used to predict occlusal wear based on sex, age, numeric rating scale/pain in the masseter, sternocleidomastoid, temporalis, and pterygoid muscles, anxiety, and stress. All these variables were found to be statistically insignificant at *F* (12, 25) = 0.807, *p*  < 0.641, *R* ^2^ = 0.279, except the numeric rating scale score of masseters (*p*  < 0.048) (Table S2: Correlation of occlusal wear (score) with viscoelastic properties (frequency, stiffness, decrement, relaxation, and creep [for each muscle of mastication]).

## 4. Discussion

The current study evaluated the changes in the viscoelastic properties of the muscles of mastication and the severity of occlusal wear in individuals with HNP. This exploratory study was based on the concept that a viscous cycle may exist between occlusion, TMJ disorders, masticatory muscle pain, and HNP. A multifactorial and complex interaction exists between peripheral, central, and psychosocial processes. When teeth are worn or the occlusal table is altered, the vertical bite decreases and the chewing cycle increases, which puts additional stress on the muscles of mastication and the TMJ [2]. When the occlusion changes, the muscles of mastication are strained to adapt to the new centric occlusion, resulting in soreness and fatigue. Occlusal wear reduces the time of the mastication cycle and increases the number of mastication cycles, which alters the overall function of the jaw muscles [37]. Sustained muscle contraction may reduce local blood flow, increase metabolic demand, and promote the accumulation of inflammatory mediators, thereby contributing to altered viscoelastic properties of muscles and increased muscle stiffness, tenderness, and pain sensitivity. Alterations in muscle viscoelastic properties may also reflect underlying changes in muscle architecture, including increased connective tissue content, changes in muscle fiber recruitment patterns, and altered neuromuscular control. These adaptations may result in elevated muscle tone and stiffness, which have been associated with myofascial pain conditions, impaired muscle function, and altered masticatory movements.

As activation of the masticatory muscles alters the way we bite, this may lead to asymmetrical loading of occlusal forces on the teeth and associated craniofacial structures. Asymmetrical loading has been shown to affect the TMJ and increase the activation of the muscle of mastication, particularly the temporalis muscle [51]. The overloading of these muscles often manifests as fatigue, pain, and soreness in the jaw muscles, with patients often complaining of TMJ pain, tooth pain, headaches, neck pain, ear pain, and tinnitus.

Previous neurophysiological research has also confirmed the presence of the trigeminocervical complex, where the sensory input from the trigeminal nerve (innervating the various muscles of mastication and the TMJ) converges with cervical spinal nerves supplying the neck muscles. This is linked to the onset of referral of pain between the jaw, head, and neck regions. There have been previous reports of the coexistence of masticatory muscle dysfunction, headaches (including tension‐type and migraine headaches), and neck pain [52, 53]. Taken together, these observations support a contemporary biopsychosocial model in which occlusal wear, muscle mechanical properties, psychological stress, and central pain processing interact to influence the onset and progression of HNP. Therefore, the associations observed in the present study should be interpreted within this broader multifactorial framework rather than as evidence of a direct or causal relationship.

We also found that individuals with HNP showed more occlusal wear, stiffness, and fatigue in their masticatory muscles. The pain scores for the muscles of mastication were higher in patients with HNP than in those without HNP. However, no correlation was observed between the muscles of mastication pain and occlusal wear. The frequency and stiffness values were significantly higher in patients with HNP than in those without HNP for both the masseter and sternocleidomastoid muscles. The relaxation and creep mean scores of the masseter and sternocleidomastoid muscles were lower in patients with HNP. These findings suggest a plausible link between occlusion, masticatory muscle function, and HNP.

These results are in line with previous studies that have demonstrated increased muscle tension and asymmetry in the masticatory muscles among individuals with occlusal discrepancies [41, 54]. Also, there have been studies that found that correcting occlusal imbalances reduces the electromyographic activity in the affected muscles and alleviates the symptoms of HNP [40, 55]. Recent evidence has also shown that individuals with neck pain or cervicogenic headaches have a higher prevalence of TMD, reduced jaw mobility, and increased somatic control [56]. Julià‐Sánchez et al. [2] explored the role of malocclusion on postural control by measuring the muscle tone, biomechanical properties (stiffness and elasticity), and viscoelastic properties of the masticatory and postural muscles (masseter, sternocleidomastoid, and erector spinalis longissimus). The authors found that dynamic postural control was significantly enhanced in measurements taken in individuals with no malocclusion (*p* = 0.04). The biomechanical and viscoelastic properties of postural muscles were found to be altered in the presence of a malocclusion. Malocclusion affected both the dynamic stability and biomechanical properties of the masticatory muscles. The study also found that the presence of crowding, Angle’s class III malocclusion, ongoing orthodontic treatment, midline deviation, increased overbite and overjet, and anterior open bite could significantly affect the frequency, stiffness, elasticity, and relaxation time of the muscles [2]. Lamey et al. [42] conducted a study to assess the correlation between the volume of muscles of mastication, craniofacial morphology, and bite force in patients suffering from migraine and found that individuals with migraine have a significant difference in the volume of the medial pterygoid and masseter muscles compared to those without migraine. Individuals with migraines had significantly higher maximal bite forces than those of controls. Individuals with headaches, such as migraines, had larger volumes of the masseter and medial pterygoid muscles and greater bite forces than controls, which could not be explained by any changes in craniofacial morphology [42, 57]. Marklund et al. [58] conducted a prospective clinical study and compared the frequency of headaches in those with TMJ disorder and found that females had higher odds of having headaches (odds ratio [OR] = 2.6). Self‐reported bruxism (OR = 2.3) and mandibular instability in the intercuspal position (OR = 3.2) were also associated with frequent episodes of headaches. Persistent headaches were significantly associated with mandibular instability in the intercuspal position (OR = 6.1) [58]. Troeltzsch et al. [22] also identified that the presence of any parafunctional habits (*p* = 0.001), TMJ disorders (*p* = 0.001), and overall differences of >3 mm between centric occlusion and maximum intercuspation are associated with headaches [22]. These findings support the role of occlusion in masticatory muscle dysfunction and HNP.

However, it is vital to note that there are many confounding factors, such as psychosocial stress, parafunctional behaviors (e.g., bruxism), cervical spine pathology, individual pain modulation, sleep quality and pattern, lifestyle, and habits (smoking), that can affect this association. Bruxism is identified as a potential risk factor for occlusal wear, masticatory muscle pain, and HNP. Bruxism, both sleep and awake, leads to sustained muscle contraction, accelerating attrition and increasing intramuscular pressure, leading to microtrauma, fatigue, and chronic stiffness of the masseter and temporalis muscles [59, 60]. Episodes of anxiety and stress have also been linked with increased central sensitization and bruxing tendencies, which in turn affect occlusal and masticatory muscle functions [36]. Patients with TMJ disorders commonly report the presence of clenching and grinding behavior, which correlates with orofacial pain symptoms such as jaw stiffness, occlusal discrepancy, and headaches [59]. Exposto et al. [61] found that patients with painful TMJ disorders have a high chance of reporting headaches (80.5% participants were found to have headaches at least once a month, and 13.8% participants reported headaches for over 2 weeks monthly). A higher proportion of moderate/severe psychological distress and physical symptoms was observed in patients with painful TMJ disorder than in those with pain in the TMJ (*p* = 0.016) [61]. A recent systematic review also confirmed a positive association between pain in the muscles of mastication and alterations in the mental status, such as depression, anxiety, and other stress‐related disorders. In the long term, muscular overactivity in individuals with stress mostly affects the temporalis and masseter muscles [35]. We also noted higher stress and anxiety scores (21.21 ± 5.64/19.42 ± 5.99) in the participants who reported painful muscles of mastication than in the controls (15.64 ± 8.99/12.08 ± 6.55); however, the results were not statistically significant.

Similarly, the presence of parafunctional habits such as pencil or pen biting, nail biting, unilateral chewing, and gum chewing increased in individuals under stress, anxiety, and other psychological disorders. This could add to existing occlusal wear and increase muscle tension in the masticatory apparatus [62, 63]. Stress is a well‐established activator of the “hypothalamic–pituitary–adrenal axis” and “sympathetic nervous system” that leads to increased production of cortisol. Elevated levels of cortisol lead to sustained muscle tension and impaired modulation of pain. The use of certain medications, such as anxiolytics, antidepressants, and selective serotonin reuptake inhibitors, has been associated with bruxism, headaches, and altered neuromuscular activity of the masticatory muscles [64]. We must also acknowledge the age‐related changes in occlusion as a nonmodifiable confounder. Tooth wear naturally increases over time, and this may be accompanied by alterations in muscle composition, reduced muscle elasticity, and changes in neuromuscular coordination, potentially affecting masticatory muscle performance [65]. Progressive occlusal flattening and compensatory mandibular adaptations may change muscle recruitment patterns in older adults, which may alter mastication and the functioning of the properties of the masticatory muscles.

Although the role of occlusion in masticatory muscle dysfunction and headaches is positively associated by many studies, and some studies have even reported improvements in masticatory muscle pain, mandibular function, and headache symptoms following occlusal splint therapy, there is recent evidence that refutes this link and has also reported limited or no superiority of occlusal interventions compared with usual care or control treatments for headache outcomes. For example, a randomized clinical trial by Saha et al. [66] reported that occlusal splint therapy was not superior to usual care in reducing the intensity of headaches among patients with chronic headache and associated TMJ disorders, highlighting the ongoing controversy regarding the clinical significance of occlusal interventions for headache management. A meta‐analysis in 2025 also reported that “occlusal stabilization appliances may provide benefit in some individuals; the overall certainty of evidence remains low, and treatment effects on headache outcomes are inconsistent” [67]. There have also been a few studies where no significant difference in maxillary and mandibular tooth wear in relation to headaches could be established [68]. Many authors have even suggested that occlusal factors alone are insufficient to explain the development of cervicogenic pain disorders. Although TMJ disorders, bruxism, headache, and cervical pain frequently coexist, the direction and causality of these relationships remain debatable. The available evidence is conflicting regarding whether these relationships are causal or merely reflect shared risk factors and overlapping pathophysiological mechanisms [69].

Collectively, these findings suggest that occlusal factors may contribute to symptom expression in some individuals but are unlikely to represent the sole etiological mechanism. Contemporary evidence increasingly supports a multifactorial model involving biomechanical, neuromuscular, behavioral, psychosocial, and cervical musculoskeletal factors in the development of pain in the muscles of mastication, TMJ, and head and neck symptoms. Hence, to better characterize how alterations in occlusion affect masticatory muscle function, the temporal sequence from peripheral dysfunction to central changes and the mediating role of various confounders, such as lifestyle, stress, and anxiety, on HNP and cervical muscle activity, should be noted. However, one must note that most of the existing evidence exploring these associations is observational in nature and does not necessarily establish causality and that the contribution of occlusal factors remains a subject of ongoing debate. Rather, occlusal wear may represent one component within a complex network of neuromuscular, occlusal, behavioral, and psychosocial factors contributing to craniofacial and cervical pain conditions.

The current study lays the foundation for future work and indicates that occlusion and masticatory muscle dysfunction could possibly be linked to HNP. Therefore, the management of headaches and pain in the muscles of mastication can include a thorough evaluation of occlusion. Dentists can be consulted to note the severity of occlusal wear, the presence of occlusal interference, and the altered occlusal pattern that may be linked with neck pain, headaches, and masticatory muscle pain. Early identification and monitoring of occlusal wear should be integrated into routine dental checkups to prevent progression that may contribute to altered viscoelastic muscle properties and headache/neck pain. Dental treatment, particularly restorative and prosthetic treatment, must prioritize maintaining or restoring harmonious occlusion to avoid exacerbating muscle stiffness and dysfunction, which can trigger or worsen HNP. Patients must be educated and informed on the role of sleep, stress, anxiety, and parafunctional habits (e.g., bruxism, clenching) and their impact on occlusal wear and muscle health and how they can contribute to HNP. Stress and anxiety management should be considered part of preventive strategies since psychosocial factors influence muscle function and occlusal wear progression. Targeted physiotherapies aimed at reducing muscle stiffness and improving relaxation times (e.g., manual therapy, myofascial release, and neuromuscular reeducation) may help patients with HNP and improve the functioning of muscles [70–72]. Thus, a multidisciplinary approach should be adopted to diagnose and treat disorders of the muscles of mastication and HNP.

Although this study found a link between occlusion, headache, and changes in the viscoelastic properties of the masticatory muscle, it is crucial to note that this study has some limitations. First, the study was conducted as a pilot cross‐sectional design with a small sample size and in a single center. This may have limited statistical power to detect smaller associations and restrict the generalizability of the findings to broader populations. Moreover, we did not include sleep quality, bruxism, or physical activity levels in the matching of groups. Furthermore, headache symptoms were recorded from participant‐reported histories, which may have been subject to a recall bias. Future studies are warranted to classify headaches and adopt specific diagnostic criteria for headache subtypes. Therefore, the HNP variable should be interpreted as the self‐reported presence of head and/or neck pain symptoms rather than a clinician‐confirmed diagnosis. The absence of standardized diagnostic criteria may have resulted in outcome heterogeneity. Lastly, we would like to add that although MyotonPro measurements were performed using a standardized protocol by a trained examiner for all participants, the assessment of muscle viscoelastic properties may be influenced by factors such as probe positioning, tissue thickness, and anatomical variability of the muscle in each participant. Hence, future longitudinal studies with larger multicenter cohorts, standardized headache diagnostic criteria, and comprehensive assessments of potential confounders are warranted.

## 5. Conclusion

Within the limitations of this pilot cross‐sectional study, participants with greater occlusal wear demonstrated altered viscoelastic properties of selected masticatory and cervical muscles and reported a higher frequency of HNP symptoms. Those with HNP had higher stress and anxiety scores than those without HNP. Although significant differences in muscle properties were observed between groups with varying degrees of occlusal wear, no statistically significant correlations were identified between occlusal wear scores and the measured viscoelastic parameters. These findings suggest a potential link exists between occlusal wear, muscle characteristics, and pain‐related outcomes; however, based on our results, causality cannot be inferred. Further large‐scale longitudinal studies with more diverse populations are required to confirm these observations. However, the assessment of occlusion, TMJ, and masticatory muscle function for all patients presenting with HNP will provide a more holistic and effective management of symptoms. These findings, although they emphasize the need to evaluate occlusal wear patterns and masticatory muscle function in patients with HNP, must be weighed with caution due small sample size. Additionally, a collaborative multidisciplinary approach involving dentists, neurologists, and physiotherapists and conducting more intervention studies in this regard are essential to effectively build a stronger association and manage the complex interplay between occlusion, muscle dysfunction, and headache/neck pain.

## Author Contributions

Conceptualization: Divyansh Sinha, Komal Smriti, and Aditi Chopra. Methodology, formal analysis, investigation, resources, data curation: Divyansh Sinha, Komal Smriti, Aditi Chopra, Srikanth Gadicherla, and G. Praburaja. Software: Divyansh Sinha, Aditi Chopra, and Prajna P. Nayak. Validation: Divyansh Sinha, Prajna P. Nayak, Aditi Chopra, and Komal Smriti. Writing – original draft, writing – review and editing, visualization: Divyansh Sinha, Komal Smriti, Aditi Chopra, Srikanth Gadicherla, G. Praburaja, and Marwa Khalil. Supervision, project administration: Aditi Chopra.

## Funding

The authors received no specific funding for this work.

## Disclosure

All authors have read and approved the final version of the manuscript. Aditi Chopra had full access to all of the data in this study and takes complete responsibility for the integrity of the data and the accuracy of the data analysis.

## Ethics Statement

The study was conducted following the Helsinki Declaration of 1975 (as revised in 2000) after receiving ethical clearance from the Kasturba Medical College and Hospital Institutional Ethics Committee (IEC Number IEC 485 – 2019).

## Consent

Written consent was taken from all participants.

## Conflicts of Interest

The authors declare no conflicts of interest.

## Supporting Information

Additional supporting information can be found online in the Supporting Information section.

## Supporting information


**Supporting Information** Table S1: Correlation between occlusal wear (score) and severity of mastication muscle pain (numeric rating scale). Table S2: Correlation of occlusal wear (score) with viscoelastic properties (frequency, stiffness, decrement, relaxation, and creep) for each muscle of mastication.

## Data Availability

The data are available upon request from the corresponding author.
