# Supplementary material for: Association Between Occlusal Wear, Viscoelastic Properties, and Pain in Masticatory Muscles in Individuals With or Without Headache and Neck Pain: A Pilot Cross‐Sectional Study
Source: Int J Dent. 2026 Jul 30;2026:7677545. doi: 10.1155/ijod/7677545 (PMC13424613; doi:10.1155/ijod/7677545)
Supplement: Supplementary file 1 — Supporting Information Table S1: Correlation between occlusal wear (score) and severity of mastication muscle pain (numeric rating scale). Table S2: Correlation of occlusal wear (score) with viscoelastic properties (frequency, stiffness, decrement, relaxation, and creep) for each muscle of mastication. [file IJOD-2026-7677545-s001.docx]

**Supplementary Table 1: Correlation of occlusal wear (score) with severity of muscle of mastication pain (numeric rating scale).**

|  | | occlusal_wear | NRS_massetter | NRS_temporalis | NRS_SCM | NRS_Pterygoid |
| --- | --- | --- | --- | --- | --- | --- |
| occlusal_wear | Pearson Correlation | 1 | -.117 | -.181 | -.185 | -.101 |
|  | Sig. (2-tailed) |  | .483 | .276 | .266 | .545 |
|  | N | 38 | 38 | 38 | 38 | 38 |
| NRS_massetter | Pearson Correlation |  | 1 | .628** | .233 | .120 |
|  | Sig. (2-tailed) |  |  | .000 | .160 | .474 |
|  | N |  | 38 | 38 | 38 | 38 |
| NRS_temporalis | Pearson Correlation |  |  | 1 | .513** | .358* |
|  | Sig. (2-tailed) |  |  |  | .001 | .027 |
|  | N |  |  | 38 | 38 | 38 |
| NRS_SCM | Pearson Correlation |  |  |  | 1 | .172 |
|  | Sig. (2-tailed) |  |  |  |  | .301 |
|  | N |  |  |  | 38 | 38 |
| NRS_Pterygoid | Pearson Correlation |  |  |  |  | 1 |
|  | Sig. (2-tailed) |  |  |  |  |  |
|  | N |  |  |  |  | 38 |
| **. Correlation is significant at the 0.01 level (2-tailed). | | | | | | |
| *. Correlation is significant at the 0.05 level (2-tailed). | | | | | | |

**Supplementary Table 2: Correlation of occlusal wear (score) with viscoelastic properties (Frequency, stiffness, decrement, relaxation, creep (for each muscle of mastication)**

|  | | **Occlusal_wear** | **Temporalis Frequency** | **Temporalis stiffness** | **Temporalis decrement** | **Temporalis relaxation** | **Temporalis creep** |
| --- | --- | --- | --- | --- | --- | --- | --- |
| **Occlusal**  **wear** | Pearson Correlation | 1 | .000 | .029 | -.040 | -.021 | -.021 |
|  | Sig. (2-tailed) |  | .999 | .869 | .821 | .904 | .908 |
|  | N | 38 | 34 | 34 | 34 | 34 | 34 |
| **Temporalis_Frequency** | Pearson Correlation |  | 1 | .886** | .127 | -.896** | -.879** |
|  | Sig. (2-tailed) |  |  | .000 | .474 | .000 | .000 |
|  | N |  | 34 | 34 | 34 | 34 | 34 |
| **Temporalis_stiffness** | Pearson Correlation |  |  | 1 | -.032 | -.962** | -.953** |
|  | Sig. (2-tailed) |  |  |  | .859 | .000 | .000 |
|  | N |  |  | 34 | 34 | 34 | 34 |
| **Temporalis_decrement** | Pearson Correlation |  |  |  | 1 | -.001 | .053 |
|  | Sig. (2-tailed) |  |  |  |  | .994 | .766 |
|  | N |  |  |  | 34 | 34 | 34 |
| **Temporalis_relaxation** | Pearson Correlation |  |  |  |  | 1 | .994** |
|  | Sig. (2-tailed) |  |  |  |  |  | .000 |
|  | N |  |  |  |  | 34 | 34 |
| **Temporalis_creep** | Pearson Correlation |  |  |  |  |  | 1 |
|  | Sig. (2-tailed) |  |  |  |  |  |  |
|  | N |  |  |  |  |  | 34 |
| ****. Correlation is significant at the 0.01 level (2-tailed).** | | | | | | | |

|  | | **Occlusal wear** | **Masseter Frequency** | **Masseter Stiffness** | **Masseter decrement** | **Masseter Relaxation** | **Masseter Creep** |
| --- | --- | --- | --- | --- | --- | --- | --- |
| **occlusal wear** | Pearson Correlation | 1 | -.276 | -.234 | .023 | .248 | .257 |
|  | Sig. (2-tailed) |  | .115 | .183 | .898 | .157 | .142 |
|  | N | 38 | 34 | 34 | 34 | 34 | 34 |
| **Masseter Frequency** | Pearson Correlation |  | 1 | .910** | .386* | -.927** | -.928** |
|  | Sig. (2-tailed) |  |  | .000 | .024 | .000 | .000 |
|  | N |  | 34 | 34 | 34 | 34 | 34 |
| **Masseter Stiffness** | Pearson Correlation |  |  | 1 | .536** | -.869** | -.859** |
|  | Sig. (2-tailed) |  |  |  | .001 | .000 | .000 |
|  | N |  |  | 34 | 34 | 34 | 34 |
| **Masseter decrement** | Pearson Correlation |  |  |  | 1 | -.463** | -.410* |
|  | Sig. (2-tailed) |  |  |  |  | .006 | .016 |
|  | N |  |  |  | 34 | 34 | 34 |
| **Masseter Relaxation** | Pearson Correlation |  |  |  |  | 1 | .998** |
|  | Sig. (2-tailed) |  |  |  |  |  | .000 |
|  | N |  |  |  |  | 34 | 34 |
| **Masseter_Creep** | Pearson Correlation |  |  |  |  |  | 1 |
|  | Sig. (2-tailed) |  |  |  |  |  |  |
|  | N |  |  |  |  |  | 34 |
| ****. Correlation is significant at the 0.01 level (2-tailed).** | | | | | | | |
| ***. Correlation is significant at the 0.05 level (2-tailed).** | | | | | | | |

|  | | **Occlusal_wear** | **Sternocleidomastoid Frequency** | **Sternocleidomastoid Stiffness** | **Sternocleidomastoid decrement** | **Sternocleidomastoid Relaxation** | **Sternocleidomastoid Creep** |
| --- | --- | --- | --- | --- | --- | --- | --- |
| **occlusal wear** | Pearson Correlation | 1 | .266 | .261 | -.195 | -.262 | -.293 |
|  | Sig. (2-tailed) |  | .129 | .136 | .270 | .135 | .093 |
|  | N | 38 | 34 | 34 | 34 | 34 | 34 |
| **Sternocleidomastoid Frequency** | Pearson Correlation |  | 1 | .938** | -.515** | -.925** | -.893** |
|  | Sig. (2-tailed) |  |  | .000 | .002 | .000 | .000 |
|  | N |  | 34 | 34 | 34 | 34 | 34 |
| **Sternocleidomastoid Stiffness** | Pearson Correlation |  |  | 1 | -.479** | -.880** | -.840** |
|  | Sig. (2-tailed) |  |  |  | .004 | .000 | .000 |
|  | N |  |  | 34 | 34 | 34 | 34 |
| **Sternocleidomastoid decrement** | Pearson Correlation |  |  |  | 1 | .480** | .542** |
|  | Sig. (2-tailed) |  |  |  |  | .004 | .001 |
|  | N |  |  |  | 34 | 34 | 34 |
| **Sternocleidomastoid Relaxation** | Pearson Correlation |  |  |  |  | 1 | .983** |
|  | Sig. (2-tailed) |  |  |  |  |  | .000 |
|  | N |  |  |  |  | 34 | 34 |
| **Sternocleidomastoid Creep** | Pearson Correlation |  |  |  |  |  | 1 |
|  | Sig. (2-tailed) |  |  |  |  |  |  |
|  | N |  |  |  |  |  | 34 |
| **. Correlation is significant at the 0.01 level (2-tailed). | | | | | | | |
